# Risk factors of recurrent erysipelas in adult Chinese patients: a prospective cohort study

**DOI:** 10.1186/s12879-020-05710-3

**Published:** 2021-01-07

**Authors:** Ang Li, Ni Wang, Lingzhi Ge, Hongyan Xin, Wenfei Li

**Affiliations:** 1 Department of Orthopaedic Surgery, Shanghai Jiao Tong University Affiliated Sixth People’s Hospital, 600 Yishan Road, Shanghai, 200233 China; 2 Department of Dermatology, The First Affiliated Hospital of Shandong First Medical University, Shandong Provincial Qianfoshan Hospital, 16766 Jingshi Road, Jinan, 250014 China; 3 Department of Dermatology, The Second Affiliated Hospital of Shandong First Medical University, 706 Taishan Street, Tai’an, 271000 China; 4 Department of Surgery, Shandong Chest Hospital, 46 Lishan Road, Jinan, 250013 China

**Keywords:** Erysipelas, Recurrent, Risk factor, Prospective, Cohort study

## Abstract

**Background:**

Erysipelas is a common skin infection that is prone to recur. Recurrent erysipelas has a severe effect on the quality of life of patients. The present study aimed to investigate the risk factors of recurrent erysipelas in adult Chinese patients.

**Methods:**

A total of 428 Chinese patients with erysipelas who met the inclusion criteria were studied. The patients were divided into the nonrecurrent erysipelas group and the recurrent erysipelas group. Clinical data were collected on the first episode and relapse of erysipelas. The patients were followed up every 3 months. Statistical analysis was performed to analyze and determine the risk factors of erysipelas relapse.

**Results:**

Univariate analysis was performed to analyze the data, including surgery, types of antibiotics administered in the first episode, obesity, diabetes mellitus, venous insufficiency, lymphedema, and malignancy. The differences between the groups were statistically significant (*p <* 0.05). The Cox proportional hazards regression model analysis showed that the final risk factors included surgery, obesity, diabetes mellitus, venous insufficiency, and lymphedema.

**Conclusions:**

Surgery, obesity, diabetes mellitus, venous insufficiency, and lymphedema are considered as risk factors for recurrent erysipelas.

## Background

Erysipelas is an acute inflammation of the skin and its reticular lymphatic vessels, which is prone to recur in the lower extremities, face, and other anatomical areas. It is frequently caused by β-hemolytic streptococci group A (*Streptococcus pyogenes*) and rarely caused by streptococci groups B, C, or G [1, 2]. The following characteristics differentiate erysipelas from other forms of tissue infections: (1) an acute, warm, slightly painful, bright red erythema with a shiny surface and a sharply defined margin as well as tongue-shaped processes, usually located a few centimeters away from the entry site; (2) a systemic inflammatory response immediately from the onset, marked by fever or at least shivering and rarely chills; and (3) increased erythrocyte sedimentation rate (ESR), increased C-reactive protein (CRP) level, and/or neutrophilia [3].

Acute and recurrent erysipelas can be differentially diagnosed on the basis of the following aspects: First, if the disease recurs in the original site, then it is termed as recurrent erysipelas; however, recurrence is not considered if two episodes of erysipelas occur in different anatomical areas in the same patient. Second, a patient with recurrent erysipelas could recover naturally in a few days, but the disease could recur within the next weeks or even years after recovery. Third, the duration of recurrent erysipelas is longer than that of acute erysipelas. Therefore, it is very important to take preventive measures to avoid the recurrence of erysipelas.

Some risk factors might be related to the recurrence of erysipelas [4]. The present article discusses the risk factors of recurrent erysipelas and provides the theoretical basis for preventing the recurrence of erysipelas.

## Methods

The present study included a prospective cohort of Chinese patients suffering from the first episode of erysipelas. The study patients were mostly residents of Shandong province, and a few patients were Chinese residents from other provinces and cities in China. A total of 525 patients with erysipelas were hospitalized in our hospital from June 2014 to June 2019, and a total of 428 inpatients with erysipelas who met the inclusion criteria were studied. The study was formally approved by the ethics committee of the First Affiliated Hospital of Shandong First Medical University.

Because β-hemolytic streptococci are sensitive to penicillin, many patients with erysipelas were administered penicillin for 7–14 days. If the patient was allergic to penicillin, clindamycin or clarithromycin or moxifloxacin was administered [3]. Other useful treatment measures included keeping the infected area elevated, placing cooling packs on the infected skin area, and applying a prescribed cream on the lesion. When the signs and symptoms subsided and laboratory biochemical markers recovered to normal after treatment, erysipelas was cured. The cohort was followed up every 3 months after being cured and discharged to observe the presence of recurrent erysipelas. All clinical data were obtained through interview, examinations, medical record, home visit, and telephone inquiry.

The diagnosis of erysipelas was based on the signs, symptoms, and results of laboratory biochemical markers consistent with the characteristics of erysipelas. The diagnosis of recurrent erysipelas was based not only on the signs, symptoms, and results of laboratory biochemical markers of erysipelas, but also on the lesions occurring at the same anatomical site as the primary erysipelas.

The patients were divided into the nonrecurrent erysipelas (NRE) group and the recurrent erysipelas (RE) group. NRE was defined as no recurrence of erysipelas, and RE was defined as one or more episodes of erysipelas during follow-up. All patients met the following inclusion criteria: (i) diagnosed to have erysipelas, (ii) over 18 years of age, (iii) no history of previous episodes of erysipelas, (iv) no other skin and soft tissue infections, (v) availability of complete information of risk factors influencing recurrent erysipelas, and (vi) available for follow-up every 3 months after being cured and discharged.

The following potential risk factors for erysipelas relapse were identified and investigated in the present study: age, sex, seasons, anatomical areas, clinical types (vulgaris type and other types), systemic symptoms, disruption of skin barrier (tinea pedis, other dermatoses, and insect bites), surgery, types of antibiotics administered, obesity (BMI ≥ 28) [5], diabetes mellitus, venous insufficiency, lymphedema, cardiovascular diseases, cerebrovascular diseases, lung diseases, liver and kidney diseases, malignancy, and autoimmune diseases. For insect bite reaction, it is manifested as papules, edematous erythema, or blisters on the bite. A small bump frequently accompanied with itching and redness develop on the skin after insect bite. Uric acid arthritis manifests as hyperuricemia. The affected joint shows severe pain, and the first joint often involves the first metatarsophalangeal joint. The characteristics of the above-mentioned diseases can be distinguished from those of primary erysipelas. In addition, stasis dermatitis is a chronic skin disease of the lower limbs that manifests as scaly, itchy skin with lymphedema, which often leads to dark brown skin pigmentation. It is caused by varicose veins. Recurrent erysipelas can cause lymphatic drainage disorder, resulting in the accumulation of lymphatic fluid in the subcutaneous tissues and formation of lymphedema. From the above-mentioned description, these diseases have different mechanisms for forming lymphedema.

### Statistical analyses

Univariate analysis was performed to analyze the differences between the two groups and to determine the factors influencing erysipelas relapse. The data were analyzed with GraphPad Prism® version 8 software, and statistical significance was considered at α = 0.05. We used the ANOVA test and the χ2 or Yates’ continuity corrected χ2 test, when appropriate. Variables were selected as candidates in multivariate analysis with Cox proportional hazards regression model based on the level of significance in univariate analysis. The statistical cutoff point for entry and exclusion was 0.2 and 0.10, respectively. Multivariate analysis was performed by IBM® SPSS® Statistics version 25.

## Results

**Fig. 1:**
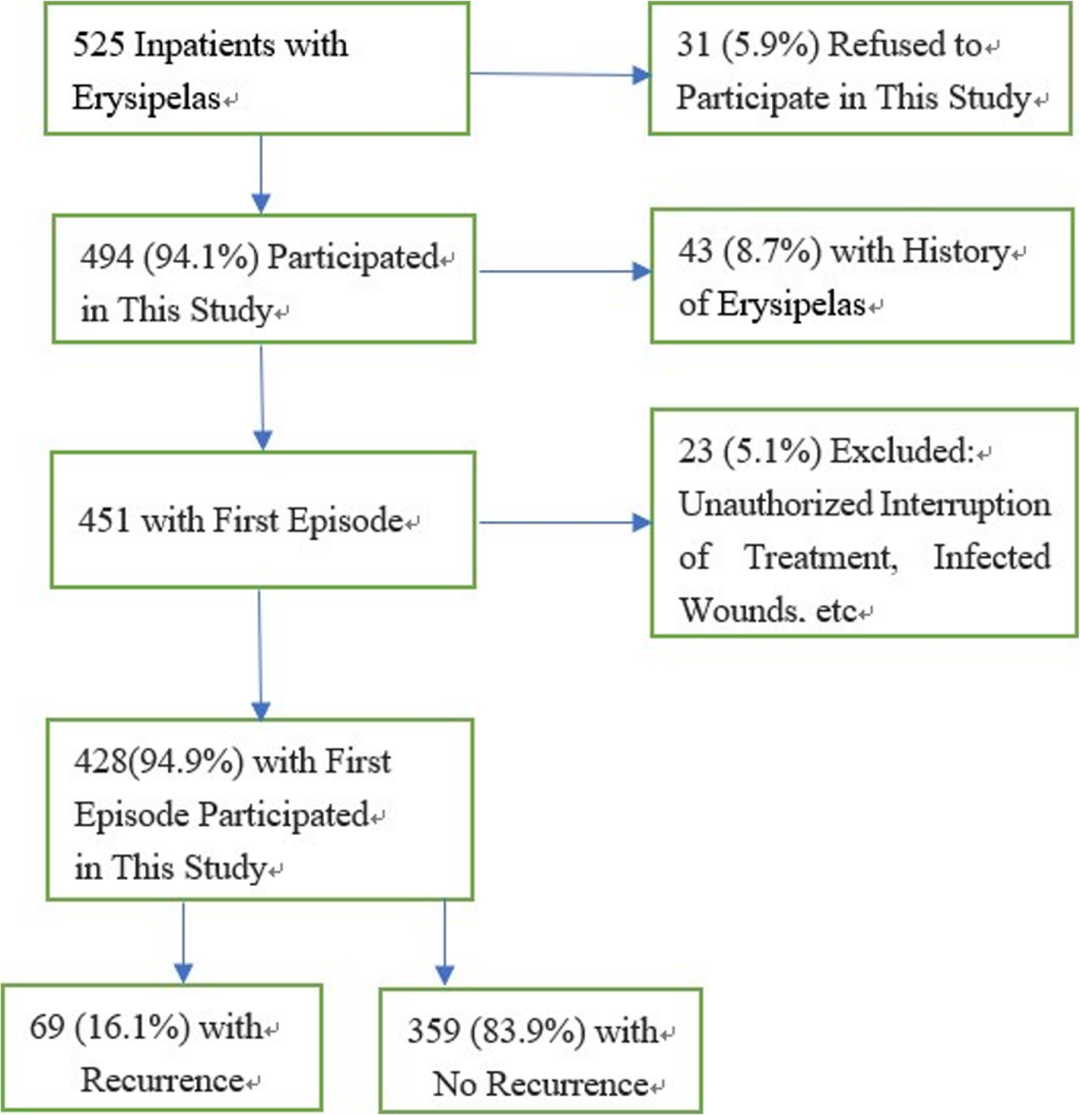
Study flowchart of risk factors for recurrence of adult inpatients with erysipelas

A total of 428 patients met the study inclusion criteria. During the follow-up, erysipelas did not recur in 359 patients and one or more episodes of erysipelas recurred in 69 patients (Fig. 1). The total number of primary and recurrent episodes was 512 in 428 patients, and 6 episodes were the greatest number of episodes in recurrent patients. The patients enrolled in this study included 231 males (54.0%) and 197 females (46.0%). The age range of the patients was 18 to 93 years. The average age of the NRE and RE groups was 57.57 ± 17.55 and 63.12 ± 15.57 years, respectively, and the difference in the average age between the two groups was statistically significant (*p* < 0.05).

**Fig. 2:**
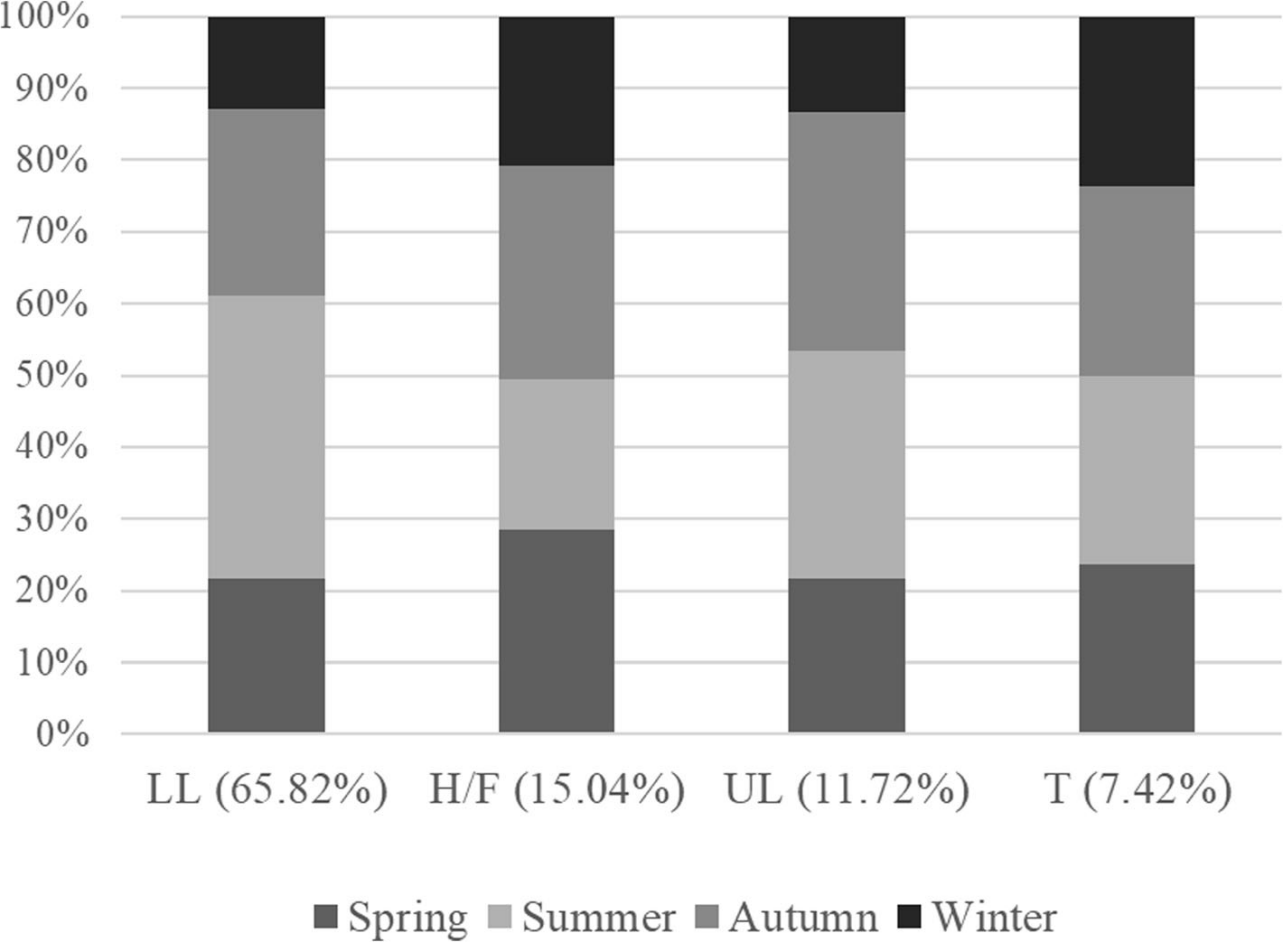
The seasons’ distribution of the first and recurrent episodes in different anatomical area. Abbreviations: LL, lower limbs; H/F, head and face; UL, upper limbs; T, trunk. Erysipelas was in lower limbs in 269 of 428 patients. Of all the episodes (*n* = 512), most episodes were in LL (65.82%), followed H/F (15.04%), UL (11.72%), T (7.42%). During the four seasons of the year, 133 (39.47%) of 337 episodes occurred in summer

Erysipelas was observed in the lower limbs (269 patients (62.85%)), followed by head/face (69 patients (16.12%)), upper limbs (54 patients (12.62%)), and trunk (36 patients (8.41%)), respectively. Most of the episodes occurred in the lower limbs (337 episodes), followed by head/face (77 episodes), upper limbs (60 episodes), and trunk (38 episodes). The season could be an initial factor for the development of recurrent erysipelas. Among the four seasons of the year, 133 of 337 episodes occurred in summer. Figure 2 shows the seasonal distribution of episodes of erysipelas in different anatomical areas.

Table 1 shows the results of univariate analysis of demographic and local risk factors. No significant differences were observed regarding gender, clinical types, systemic symptoms, and disruption of skin barrier; however, significant differences were observed regarding surgery and types of antibiotics administered in the first episode between the two groups. Univariate analysis of general risk factors related to erysipelas relapse showed that cardiovascular diseases, cerebrovascular diseases, lung diseases, liver and kidney diseases, and autoimmune diseases were not significant risk factors (*p* > 0.05). As shown in Table 2, the differences in obesity, diabetes mellitus, venous insufficiency, lymphedema, and malignancy were statistically significant (*p* < 0.05).

**Table 1:** Univariate analysis of demographic and local risk factors characteristics in the NRE group and RE group

Variable	NRE (*n* = 359)	RE (*n* = 69)	***X***^***2***^	***p*** value	OR (95%CI)
**Age**	57.57 ± 17.55	63.12 ± 15.57	–	0.02	–
**Gender**			0.215	0.64	1.1 (0.7–1.9)
male	167 (46.5)	30 (43.5)			
female	192 (53.5)	39 (56.5)			
**Anatomical areas**			9.01	0.03	
lower limbs	215 (59.9)	54 (78.3)			1 (reference)
head/face	61 (17.0)	8 (11.6)			0.5 (0.2–1.2)
upper limbs	49 13.6)	5 (7.2)			0.4 (0.2–1.0)
trunk	34 (9.5)	2 (2.9)			0.2 (0.1–0.9)
**Clinical types**			0.005	0.94	0.9 (0.3–2.8)
vulgaris type	341 (95.0)	66 (95.7)			
other types	18 (5.0)	3 (4.3)			
**Systemic symptoms**	147 (40.9)	28 (40.6)	0.003	0.95	1.0 (0.6–1.7)
**Disruption of Skin barrier**	45 (12.5)	11 (15.9)	0.591	0.44	1.3 (0.6–2.7)
**Surgery**	62 (17.3)	32 (46.4)	28.61	< 0.0001	4.1 (2.4–7.1)
**Antibiotic varieties for first episode**			28.04	< 0.0001	5.0 (2.6–9.3)
penicillin	330 (91.9)	48 (69.6)			
other classes of antibiotics	29 (8.1)	21 (30.4)			

Systemic symptoms: including high fever, chills, headache, muscle ache, fatigue, nausea, vomiting

Disruption of skin barrier: including tinea pedis, other dermatosis and insect bites

**Table 2:** Univariate analysis of comorbidities – general risk factors in the NRE group and RE group

Variable	NRE (*n* = 359)	RE (*n* = 69)	***X***^***2***^	***p*** value	OR (95%CI)
Obesity^a^	62 (17.3)	28 (40.6)	18.94	< 0.0001	3.3 (1.9–5.7)
Diabetes mellitus^b^	47 (13.1)	16 (23.2)	4.70	0.03	2.0 (1.0–3.8)
Cardiovascular diseases	86 (24.0)	15 (21.7)	0.16	0.69	0.9 (0.5–1.7)
Cerebrovascular diseases	48 (13.4)	10 (14.5)	0.06	0.80	1.1 (0.5–2.3)
Lung diseases	37 (10.3)	8 (11.6)	0.10	0.75	1.1 (0.5–2.5)
Liver and kidney diseases	34 (9.47)	7 (10.1)	0.03	0.86	1.1 (0.4–2.5)
Venous insufficiency	20 (5.6)	16 (23.2)	23.32	< 0.0001	5.1 (2.5–10.7)
Lymphedema	21 (5.85)	18 (26.1)	28.62	< 0.0001	5.7 (2.7–11.4)
Malignancy	20 (5.57)	9 (13.0)	5.12	0.02	2.5 (1.1–5.8)
Autoimmune diseases	8 (2.2)	3 (4.3)	1.04	0.31	2.0 (0.6–6.7)

^a^Obesity, body mass index ≥28, ^b^Diabetes mellitus, Type 2, expect for one patient with type 1

The Cox proportional hazards regression model was used to analyze the risk factors selected from univariate analysis. The analysis showed that surgery, obesity, diabetes mellitus, venous insufficiency, and lymphedema were risk factors for the recurrence of erysipelas (Table 3).

**Table 3:** Cox proportional hazards regression model analysis of the correlation between erysipelas recurrence and risk factors

Variable	***P*** value	HR	95%CI
surgery	< 0.0001	3.527	2.410–5.162
obesity	< 0.0001	27.298	16.543–24.044
diabetes mellitus	< 0.0001	7.693	5.153–11.487
Venous insufficiency	< 0.0001	4.020	2.473–6.534
lymphedema	< 0.0001	3.821	2.324–6.283

*Abbreviations*: *HR* hazard ratio, *CI* confidence interval

## Discussion

Erysipelas is a bacterial infectious disease with a tendency to recur. Its recurrence may lead to skin damage, scars, and chronic lymphedema, eventually resulting in the development of elephantiasis, which poses a risk to people’s health and increases medical costs and social burden [6]. Therefore, it is crucial to understand the risk factors for erysipelas recurrence in order to control and prevent it. However, to the best of our knowledge, few studies have been conducted on the risk factors for the recurrence of erysipelas. The present study is the first to report the risk factors for the recurrence of erysipelas in adult Chinese patients in English literature.

Several studies have suggested that erysipelas has a high recurrence rate and that the recurrence of erysipelas could be related to some potential risk factors [7]. The recurrence rate in the present study was 16.12%, which was lower than that reported in a previous study [6]. The reason for this difference may be related to differences in race, living environment, etc. Moreover, the criteria used for erysipelas have not always been so clearly defined in previous case series or other studies so that some of them may have encompassed also S.aureus-mediated soft tissue infections (sometimes referred to as cellulitis) which have less tendency to relapse. However, the new diagnostic criteria for erysipelas will have a significant impact on the distinction between erysipelas and cellulitis, and the recurrence rate of erysipelas [3].

The average age of the patients in the present study was relatively old, suggesting that many patients with erysipelas were elderly people. They might be less resistant to infections and had more complications, which were the risk factors for the occurrence and recurrence of erysipelas. Hence, older people show more willingness than young people to be hospitalized for treating erysipelas [8].

In the present study, erysipelas was observed in lower limbs in 269 patients (*X*^*2*^ = 9.01, *p* = 0.03 vs. other anatomical areas). Of the 512 episodes, most episodes (65.82%) occurred in the lower limbs. Furthermore, of the 337 episodes of erysipelas that occurred in the lower limbs, 133 episodes (39.47%) occurred in summer. Therefore, summer was the season of high incidence of erysipelas.

Univariate analysis showed no significant differences in sex, clinical types, systemic symptoms, and skin barrier disruption between the two groups. These results indicated that these factors were not closely associated with the recurrence of erysipelas. Tinea pedis, dermatoses, and insect bites can disrupt the skin barrier and cause skin infections; hence, the incidence of erysipelas in the lower limbs is higher, especially in summer [9]. However, because these factors caused mild and temporary damage of the skin barrier, they did not seem to be the risk factors for erysipelas relapse [10]. Surgery can change the local tissue structure and cause poor lymphatic reflux [*p* < 0.0001, OR 4.1 (95% CI 2.4–7.1)] [11]. The results of univariate analysis further indicated that surgery was not the only risk factor for erysipelas, but there were other risk factors as well. We also found that the types of antibiotics administered in the first episode were related to erysipelas recurrence [*p* < 0.0001, OR 5.0 (95% CI 2.6–9.3)]. The recurrence rate after penicillin administration was lower than that after the administration of macrolides, quinolones, and aminoglycosides. The reason for this difference may be that most of the pathogens that cause erysipelas were more sensitive to penicillin and that the clearance rate of penicillin was higher; hence, the clinical effect was better [12].

Univariate analysis showed no significant differences in the incidence of cardio-cerebral-vascular diseases, lung diseases, and liver and kidney diseases between the two groups; this finding suggested that these factors were not directly related to the recurrence of erysipelas. Obesity not only affects blood pressure and venous circulation in the lower limbs, but it also causes insulin resistance, which might lead to diabetes. A sustained increase in blood glucose concentration can cause dysfunction of mononuclear cells, reduce the body’s resistance to pathogens, and thus increase the susceptibility of the patient to easily develop infection [13]. Therefore, obesity [*p* < 0.0001, OR: 3.3 (95% CI: 1.9–5.7)] and diabetes [*P* = 0.03, OR: 2.0 (95% CI: 1.0–3.8)] can increase the probability of developing infection and lead to the recurrence of erysipelas. Our present study confirmed that malignancy [*p* = 0.02, OR: 2.5 (95% CI:1.1–5.8)], venous insufficiency [*p* < 0.0001, OR: 5.1 (95% CI: 2.5–10.7)], and lymphedema [*p* < 0.0001, OR: 5.7 (95% CI: 2.7–11.4)] were the possible risk factors for the recurrence of erysipelas. These factors sometimes existed independently, but they often interacted and influenced each other. Malignancy may cause patients to develop abnormal immune functions, while malignant radiotherapy may reduce leukocyte count [14]. Surgical treatment can disrupt the barrier function of the skin and affect the lymphatic circulation. For example, radical mastectomy can cause lymphedema in the surgical area [15]. Venous insufficiency can cause lower extremity edema, stasis dermatitis [16], and even ulcers. These conditions might be the risk factors for the occurrence and recurrence of erysipelas. We also analyzed these risk factors by using the Cox proportional hazards regression model. The analysis revealed that surgery, obesity, diabetes mellitus, venous insufficiency, and lymphedema were the probable risk factors for the recurrence of erysipelas.

The present study has some limitations. First, the average length of hospital stay was 12.66 ± 4.89 days, and it was longer than that noted in previous studies [4, 17]. This might be related to the medical insurance system in mainland China; in this system, a medical insurance company pays a certain percentage of medical expenses incurred only when a patient is hospitalized. Second, the C-reactive protein (CRP) level and peripheral blood white blood cell (WBC) count were not investigated. This is because only one previous study showed differences in CRP and WBC between patients with erysipelas and those with recurrent erysipelas [18], but other studies showed no such differences [12, 19]. In addition, a new study has elaborated the usefulness of CRP and differential (neutrophil) blood count for differentiating between erysipelas and S.aureus-mediated cellulitis [20]. Therefore, we will focus more on this aspect in our future studies. Third, antibiotics produced by different manufacturers may affect the clinical outcomes observed in the present study.

## Conclusion

Erysipelas is an infectious skin disease that frequently occurs in elderly people. It usually tends to recur, and surgery, obesity, diabetes mellitus, venous insufficiency, and lymphedema are the probable risk factors for its recurrence. Erysipelas most frequently occurs in the lower extremities, and its onset depends on the season. Therefore, while actively treating erysipelas, doctors should also understand the possible risk factors affecting erysipelas recurrence in order to prevent it.

## Data Availability

The datasets generated and analyzed during the current study are not publicly available since it contains personal information, butare available from the corresponding author on reasonable request.

## Abbreviations

NRE: Nonrecurrent erysipelas group
RE: Recurrent erysipelas group
CRP: C-reactive protein
WBC: White blood cell count
